# Association of Left Atrial Strain With One-Year Clinical Outcomes in Patients Hospitalized With Acute Decompensated Heart Failure With Preserved Ejection Fraction

**DOI:** 10.7759/cureus.113911

**Published:** 2026-08-03

**Authors:** Lejla Brigic, Alen Džubur, Lejla Dervišević

**Affiliations:** 1 Department of Heart Diseases, Blood Vessels and Rheumatism, Clinical Center University of Sarajevo, Sarajevo, BIH; 2 Department of Human Anatomy, Faculty of Medicine, University of Sarajevo, Sarajevo, BIH

**Keywords:** atrial myopathy, diastolic dysfunction, echocardiography, hfpef, left atrial strain, mace, pals

## Abstract

Background

In patients with heart failure with preserved ejection fraction (HFpEF), left atrial (LA) strain represents a new indicator of mechanical atrial dysfunction and an important prognostic marker. However, the clinical significance of serial changes in LA strain during patient follow-up is still not sufficiently elucidated.

Objectives

To evaluate changes between baseline and one-year follow-up in LA strain during one-year follow-up in patients hospitalized with HFpEF and to investigate their association with major adverse cardiovascular events (MACE).

Methods

This prospective observational cohort study included 123 consecutive patients hospitalized due to acute decompensated HFpEF. Comprehensive echocardiographic evaluation was performed during index hospitalization and after 12 months. LA reservoir strain was assessed as peak atrial longitudinal strain (PALS). The primary endpoint was MACE, defined as rehospitalization due to worsening heart failure and/or cardiovascular death during one-year follow-up.

Results

Patients who developed MACE had lower baseline LA strain, higher LAVI, higher E/e′, higher N-terminal pro-B-type natriuretic peptide (NT-proBNP), higher right ventricular systolic pressure (RVSP), lower tricuspid annular plane systolic excursion (TAPSE), and more impaired left ventricular global longitudinal strain (LV GLS) compared with patients without MACE. Assessment at two predefined time points showed overall improvement in LA strain during follow-up, but patients with adverse outcomes remained characterized by persistently impaired LA strain and an unfavorable hemodynamic profile. Atrial fibrillation was more frequently observed among patients who developed MACE.

Conclusion

Persistently impaired or worsening LA strain during one-year follow-up was associated with adverse outcomes in patients hospitalized with HFpEF. Assessment of LA strain at baseline and one-year follow-up may improve longitudinal risk stratification beyond conventional echocardiographic parameters.

## Introduction

Heart failure with preserved ejection fraction (HFpEF) accounts for a substantial proportion of heart failure cases and remains a major unresolved challenge in contemporary cardiology [1-4]. Despite preserved left ventricular ejection fraction (LVEF), patients with HFpEF frequently experience recurrent hospitalization, impaired exercise capacity, poor quality of life, and increased cardiovascular mortality.

Current guideline and consensus documents emphasize that HFpEF is not defined by a single echocardiographic parameter, but rather by a constellation of clinical symptoms, natriuretic peptide elevation, structural heart disease, and functional evidence of elevated filling pressures [1-4]. The contemporary concept of HFpEF has shifted from an isolated disorder of left ventricular relaxation toward a systemic syndrome involving inflammation, endothelial dysfunction, microvascular disease, myocardial fibrosis, ventricular-vascular interaction, and atrial remodeling [5,6].

The left atrium (LA) is increasingly recognized as a key component of HFpEF pathophysiology. Chronic elevation of left ventricular filling pressures promotes LA remodeling, fibrosis, impaired compliance, and mechanical dysfunction [7-10]. LA dysfunction is clinically relevant because it reflects cumulative hemodynamic burden and may contribute directly to pulmonary venous hypertension, atrial fibrillation, reduced exercise tolerance, and recurrent decompensation.

Left atrial volume index (LAVI) is widely used as a marker of chronic diastolic burden, but structural remodeling may appear later than functional impairment [11,12]. LA strain, particularly LA reservoir strain or peak atrial longitudinal strain (PALS), provides a direct assessment of atrial mechanical function and may identify atrial dysfunction before overt atrial enlargement becomes evident [13-17].

HFpEF is increasingly recognized as a heterogeneous multisystem syndrome characterized not only by diastolic dysfunction, but also by systemic inflammation, endothelial dysfunction, microvascular rarefaction, myocardial fibrosis, pulmonary vascular remodeling, and abnormal ventricular-vascular coupling [18,19]. In this contemporary paradigm, atrial remodeling and atrial mechanical dysfunction represent central pathophysiological components rather than secondary epiphenomena.

The concept of atrial myopathy has emerged as an important mechanistic substrate linking chronically elevated filling pressures, atrial fibrosis, impaired reservoir function, atrial fibrillation, pulmonary hypertension, and recurrent heart failure decompensation [20-22]. Consequently, assessment of left atrial mechanics may provide incremental information beyond conventional structural parameters such as LAVI.

While most previous studies evaluated LA strain at a single time point, HFpEF is a dynamic progressive syndrome characterized by ongoing hemodynamic and structural remodeling. Assessment at two predefined time points of LA strain may therefore provide additional insight into disease trajectory, atrial reverse remodeling, and longitudinal risk stratification.

Several studies have shown that impaired LA strain is associated with elevated filling pressures, abnormal exercise hemodynamics, pulmonary hypertension, hospitalization, and mortality in HFpEF [23-27]. More recent literature further supports the role of LA strain and atrial myopathy in HFpEF phenotyping and risk stratification [28,29]. However, most studies have evaluated LA strain at a single time point. Data regarding serial LA strain changes during longitudinal follow-up remain limited.

The aim of this study was to evaluate changes between baseline and one-year follow-up in LA strain during one-year follow-up in patients hospitalized with HFpEF and to investigate their association with adverse cardiovascular outcomes. This study specifically focused on longitudinal changes in atrial mechanical function after hospitalization for acute decompensated HFpEF, an area that remains insufficiently explored in contemporary HFpEF imaging research.

## Materials and methods

Study design and population

This prospective observational cohort study included 123 consecutive patients hospitalized with acute decompensated HFpEF at the Clinical Center University of Sarajevo between January 25, 2024, and December 31, 2024. Consecutive patients were prospectively enrolled throughout the study period to minimize selection bias. All participants underwent comprehensive baseline echocardiographic evaluation during the index hospitalization and were followed for 12 months after discharge.

The mean age of the study population was 72.2 ± 7.7 years, and female patients predominated (59.3%). Atrial fibrillation was present in 41 patients (33.3%), while the remaining patients were in sinus rhythm at the time of echocardiographic assessment.

The study was conducted in accordance with the Declaration of Helsinki and approved by the Ethics Committee of the Clinical Center University of Sarajevo, approval number 02-3-4-AK-681/24. Written informed consent was obtained from all participants.

HFpEF diagnosis required symptoms and/or signs of heart failure, LVEF ≥50%, elevated natriuretic peptide concentrations, and objective evidence of structural heart disease and/or diastolic dysfunction according to contemporary European Society of Cardiology (ESC) and American College of Cardiology/American Heart Association/Heart Failure Society of America (ACC/AHA/HFSA) recommendations.

Patients with reduced ejection fraction, severe primary valvular disease, infiltrative cardiomyopathy, congenital heart disease, or inadequate echocardiographic image quality were excluded.

Echocardiographic assessment

Standard transthoracic echocardiography was performed according to the recommendations of the American Society of Echocardiography (ASE) and the European Association of Cardiovascular Imaging (EACVI) for cardiac chamber quantification and diastolic function assessment [11,12]. All examinations were performed by an experienced certified echocardiographer in the left lateral decubitus position using a commercially available ultrasound system (Vivid S70; GE Vingmed Ultrasound, Horten, Norway) equipped with an M4S-RS phased-array transducer (1.5-3.6 MHz).

The following echocardiographic parameters were analyzed: LVEF, left ventricular global longitudinal strain (LV GLS), LAVI, E/e′ ratio, right ventricular systolic pressure (RVSP), tricuspid annular plane systolic excursion (TAPSE), and LA strain.

LV GLS was assessed using two-dimensional speckle-tracking echocardiography with vendor-specific software (EchoPAC; GE Medical Systems Ultrasound and Primary Care Diagnostics, LLC, GE HealthCare, Waukesha, WI, USA). GLS analysis was performed from standard apical two-, three-, and four-chamber views. Peak systolic longitudinal strain values from all left ventricular segments were averaged to obtain global longitudinal strain. More negative GLS values reflected better left ventricular systolic function.

Left atrial volumes were measured from apical two- and four-chamber views using the biplane method of disks, excluding the pulmonary veins, left atrial appendage, and mitral valve area. Maximal left atrial volume was measured at end-systole and indexed to body surface area (BSA) to obtain the LAVI.

Left atrial strain analysis was performed using two-dimensional speckle-tracking echocardiography with vendor-specific software (EchoPAC) [11-14]. Left atrial reservoir function was assessed as PALS obtained from apical four- and two-chamber views during ventricular systole [13,14]. In patients with sinus rhythm, measurements were averaged over three consecutive cardiac cycles, whereas in patients with atrial fibrillation, five consecutive cardiac cycles were averaged to minimize beat-to-beat variability.

All echocardiographic measurements were performed by an experienced operator according to current ASE/EACVI recommendations.

The E/e′ ratio was calculated using pulsed-wave Doppler assessment of transmitral early diastolic inflow velocity (E wave) and tissue Doppler imaging-derived early diastolic mitral annular velocity (e′). Septal and lateral e′ velocities were obtained in the apical four-chamber view, and the average E/e′ ratio was used for analysis as an estimate of left ventricular filling pressures.

Clinical outcomes

Changes in left atrial mechanical function during follow-up may provide important insight into atrial remodeling and disease progression in patients with HFpEF. Based on the presence or absence of recurrent decompensation or cardiovascular death, patients were divided into two groups: patients who experienced recurrent heart failure decompensation and/or cardiovascular death during follow-up, and patients without adverse events during the follow-up period.

Major adverse cardiovascular events (MACE) included rehospitalization due to worsening heart failure and cardiovascular death.

The primary endpoint was the composite of heart failure rehospitalization and/or cardiovascular death during one-year follow-up. Patients without either event were classified as the non-MACE group.

Patients were invited for a follow-up clinical and echocardiographic examination one year after discharge from the index hospitalization and were additionally monitored through telephone interviews and review of hospital electronic medical records using the BIS (Hospital Information System).

Statistical analysis

Statistical analysis was performed using IBM SPSS Statistics for Windows, version 25.0 (released 2017; IBM Corporation, Armonk, NY, USA). Categorical variables are expressed as numbers and percentages. Between-group comparisons were performed using independent samples t-test or nonparametric testing where appropriate. Paired comparisons between baseline and follow-up values were performed using paired testing. A receiver operating characteristic (ROC) curve analysis was used to assess discriminatory performance for MACE prediction. Kaplan-Meier analysis was performed using baseline LA strain threshold derived from the ROC analysis. A p-value <0.05 was considered statistically significant. Exploratory multivariable analysis adjusting for age, atrial fibrillation, N-terminal pro-B-type natriuretic peptide (NT-proBNP), and LAVI was additionally performed to assess the independent prognostic value of LA strain. Variables included in the multivariable model were selected based on clinical relevance and univariable association with outcomes.

Continuous variables are presented as mean ± standard deviation or median (interquartile range), according to data distribution. Categorical variables are presented as frequencies and percentages. Variables showing clinical relevance or significant univariable association with adverse outcomes were entered into the multivariable logistic regression model.

## Results

Baseline clinical and echocardiographic characteristics according to MACE

**Figure 1:**
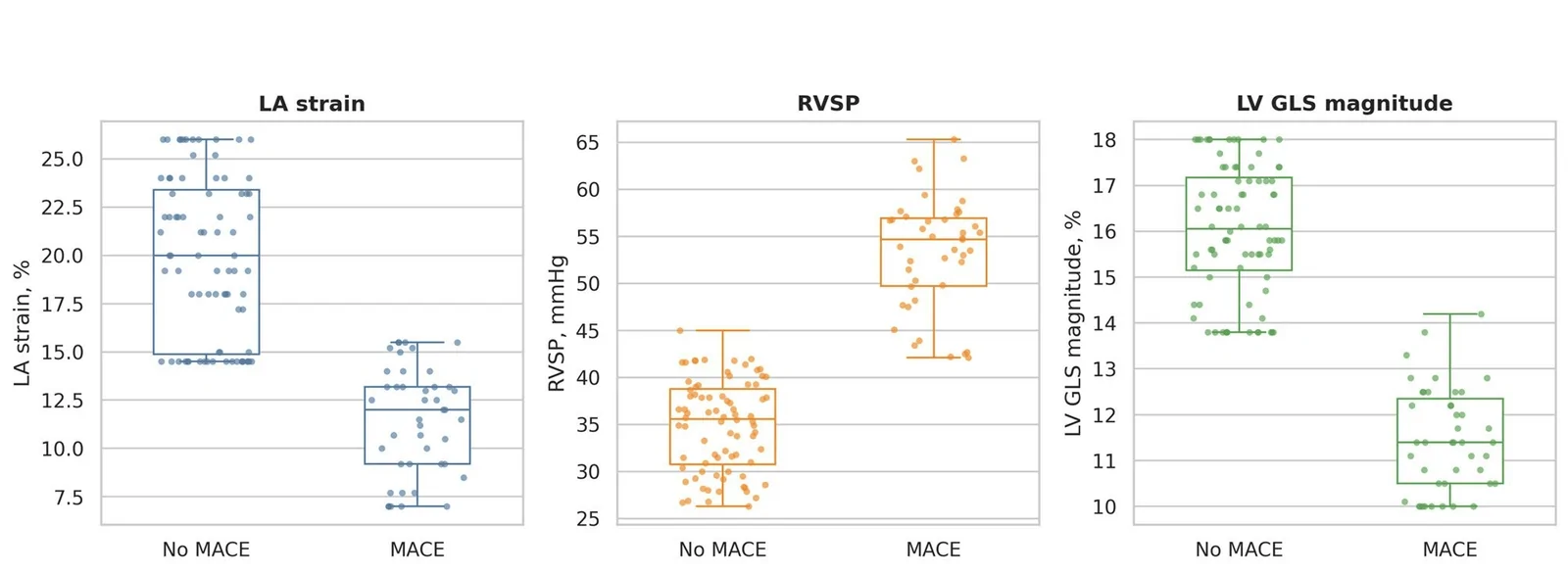
Individual-patient distributions of baseline LA strain, RVSP, and LV GLS magnitude according to MACE status. Boxes show the median and interquartile range; dots represent individual patients (MACE, n = 43; no MACE, n = 80). LA: left atrium; RVSP: right ventricular systolic pressure; LV GLS: left ventricular global longitudinal strain; MACE: major adverse cardiovascular events

Formal normality testing confirmed that baseline NT-proBNP was non-normally distributed (Shapiro-Wilk p<0.001); therefore, it is reported as median (IQR) and compared between groups using the Mann-Whitney U test. After recalculation directly from the individual-patient dataset, the independent samples t-statistics were 18.46 for RVSP, 12.26 for TAPSE, and 18.43 for LV GLS magnitude. During the 12-month follow-up, 40 patients experienced heart failure rehospitalization and 9 experienced cardiovascular death; 6 patients experienced both component events. Accordingly, the composite MACE endpoint occurred in 43 unique patients, while 80 patients experienced neither event. Atrial fibrillation was present in a substantial proportion of the study population and was more frequently observed among patients who developed MACE during follow-up, further supporting the close pathophysiological relationship between atrial mechanical dysfunction, atrial remodeling, and adverse outcomes in HFpEF. Patients who experienced MACE demonstrated markedly lower baseline LA strain compared with patients without MACE (11.5 ± 2.8% vs. 19.9 ± 4.2%, p < 0.001). Follow-up LA strain also remained substantially lower in the MACE group (Table 1, Figure 1).

**Table 1:** Baseline clinical and echocardiographic characteristics according to MACE Data are presented as mean ± SD, median (IQR), or n (%). Independent samples t-tests were used for normally distributed continuous variables, Mann-Whitney U test for NT-proBNP, and chi-square tests for categorical variables. MACE and no-MACE groups included 43 and 80 patients, respectively. SD values were recalculated directly from the individual-patient dataset. A two-sided p-value <0.05 was considered statistically significant. LVEF: left ventricular ejection fraction; LA: left atrium; LAVI: left atrial volume index; NT-proBNP: N-terminal pro-B-type natriuretic peptide; RVSP: right ventricular systolic pressure; TAPSE: tricuspid annular plane systolic excursion; LV GLS: left ventricular global longitudinal strain; MACE: major adverse cardiovascular events; SD: standard deviation; IQR: interquartile range

Variable	Overall (n=123)	MACE (n=43)	No MACE (n=80)	p-value	Test statistic
Age, years	72.0 ± 7.7	73.8 ± 8.3	71.1 ± 7.3	0.062	t = 1.89
Female sex, n (%)	74 (60.2)	24 (55.8)	50 (62.5)	0.470	χ² = 0.52
LVEF, %	55.3 ± 2.7	52.9 ± 1.8	56.6 ± 2.1	<0.001	t = 9.80
Baseline LA strain, %	16.9 ± 5.5	11.5 ± 2.8	19.9 ± 4.2	<0.001	t = 11.85
Baseline LAVI, mL/m²	65.1 ± 18.9	82.7 ± 18.1	55.6 ± 10.7	<0.001	t = 10.42
Baseline E/e′	15.0 ± 3.3	18.4 ± 2.7	13.1 ± 1.6	<0.001	t = 13.54
NT-proBNP, pg/mL	1550 (980-2690)	3700 (2500-5375)	1250 (820-1562)	<0.001	U = 3297
RVSP, mmHg	41.3 ± 10.3	53.3 ± 6.0	34.9 ± 4.8	<0.001	t = 18.46
TAPSE, mm	18.4 ± 2.8	15.5 ± 1.7	19.9 ± 1.9	<0.001	t = 12.26
LV GLS magnitude, %	14.4 ± 2.6	11.5 ± 1.1	16.0 ± 1.4	<0.001	t = 18.43
Atrial fibrillation, n (%)	50 (40.7)	27 (62.8)	23 (28.8)	<0.001	χ² = 13.43

Changes in echocardiographic parameters between baseline and one-year follow-up

Changes in echocardiographic parameters between baseline and one-year follow-up are presented in Table 2. Among 114 patients with paired measurements, LA strain increased from 17.3 ± 5.5% at baseline to 21.1 ± 7.3% after one year (p < 0.001). LAVI, E/e′, NT-proBNP, and RVSP decreased during follow-up. NT-proBNP decreased from a median of 1350 pg/mL (IQR: 980-2635) to 842 pg/mL (IQR: 551-1809; Wilcoxon p < 0.001).

**Table 2:** Changes in echocardiographic and biomarker parameters between baseline and one-year follow-up. Data are presented as mean ± SD or median (IQR). Paired-samples t-tests were used for normally distributed variables, whereas NT-proBNP was analyzed using the Wilcoxon signed-rank test. Analyses included 114 patients with complete paired measurements. A two-sided p-value <0.05 was considered statistically significant. LA: left atrium; LAVI: left atrial volume index; NT-proBNP: N-terminal pro-B-type natriuretic peptide; RVSP: right ventricular systolic pressure; LV GLS: left ventricular longitudinal strain; SD: standard deviation

Parameter	Baseline (n=114)	One-year follow-up (n=114)	Change	Paired p	Test statistic
LA strain, %	17.3 ± 5.5	21.1 ± 7.3	3.7 ± 2.7	<0.001	t = 15.00
LAVI, mL/m²	63.2 ± 17.5	61.0 ± 18.6	-2.1 ± 2.4	<0.001	t = 9.76
E/e′	14.6 ± 3.0	13.0 ± 4.2	-1.6 ± 1.6	<0.001	t = 10.58
NT-proBNP, pg/mL	1350 (980-2635)	842 (551-1809)	-443 (-736 to -254)	<0.001	W = 371
RVSP, mmHg	39.8 ± 9.1	36.9 ± 11.7	−2.9 ± 3.4	<0.001	t=9.03
LV GLS magnitude, %	14.7 ± 2.5	16.0 ± 3.1	1.3 ± 0.9	<0.001	t=16.00

LA strain and adverse outcomes

**Figure 2:**
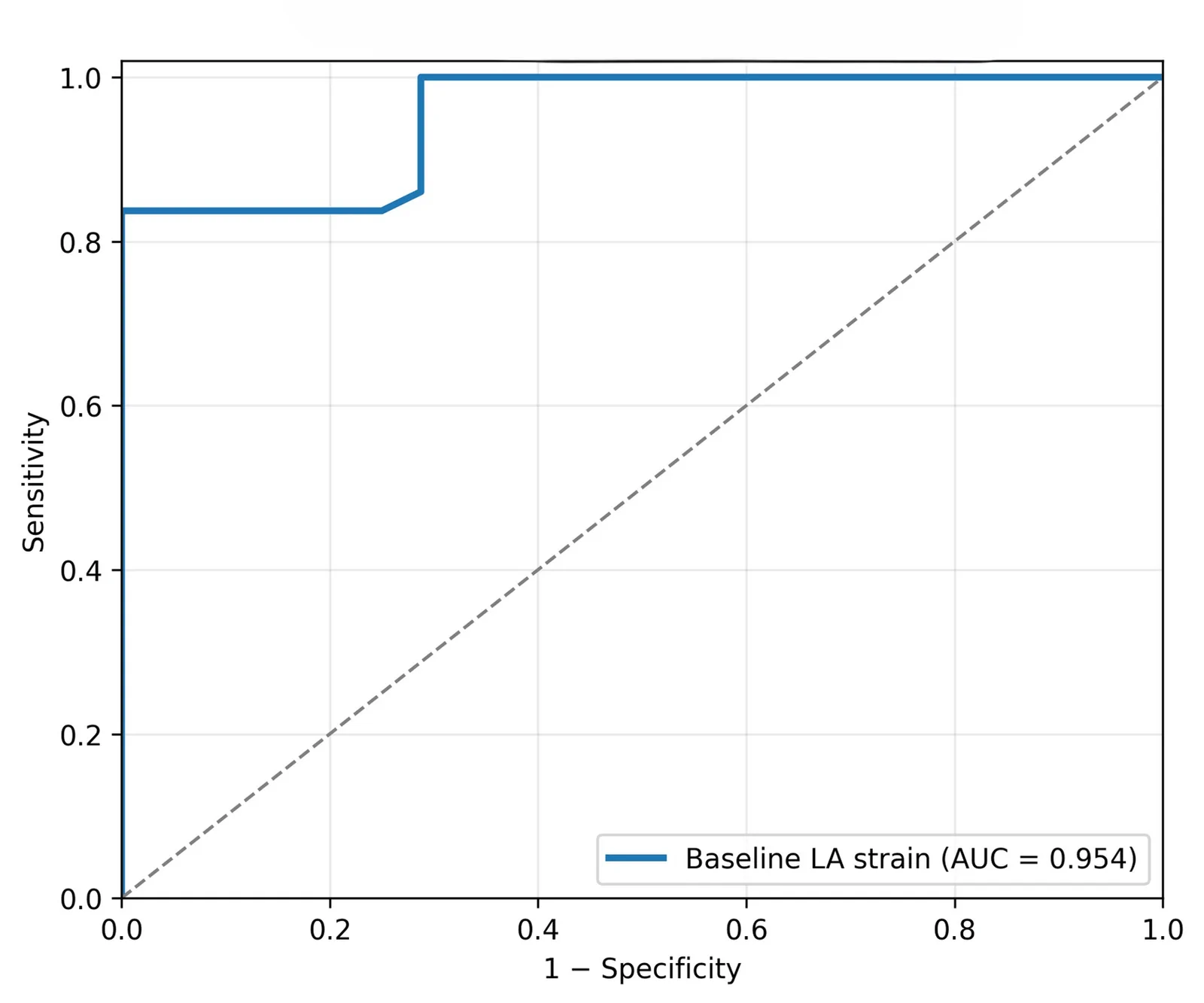
ROC curve for baseline LA strain predicting MACE ROC curve demonstrating the discriminatory performance of baseline LA strain for prediction of major adverse cardiovascular events (MACE). AUC = 0.954. LA: left atrium; ROC: receiver operating characteristic; AUC: area under the receiver operating characteristic curve

Baseline LA strain ≤14.0% showed excellent discriminatory performance for prediction of MACE, with an AUC of 0.954, sensitivity of 83.7%, and specificity of 100.0%. Follow-up LA strain and delta LA strain also showed very high discriminatory performance. Given the near-complete separation observed for several markers, these findings should be interpreted cautiously and require external validation (Table 3; Figure 2).

**Table 3:** ROC analysis for prediction of MACE. LA: left atrium; LAVI: left atrial volume index; NT-proBNP: N-terminal pro-B-type natriuretic peptide; AUC: area under the receiver operating characteristic curve

Marker	AUC	Cut-off	Sensitivity	Specificity
Baseline LA strain	0.954	14.0	83.7%	100.0%
Follow-up LA strain	0.999	16.3	97.1%	100.0%
Delta LA strain	0.996	2.4	100.0%	98.8%
Baseline LAVI	0.890	62.0	97.7%	71.2%
Baseline E/e′	0.979	15.2	90.7%	91.2%
Baseline NT-proBNP	0.958	2100.0	90.7%	88.8%

In exploratory Firth penalized multivariable analysis, baseline LA strain remained associated with adverse outcomes after adjustment for age, atrial fibrillation, NT-proBNP, and LAVI (Table 4).

**Table 4:** Firth penalized multivariable logistic regression analysis for prediction of adverse HF-related outcomes Firth penalized logistic regression was used because standard maximum-likelihood logistic regression was unstable in the presence of near-complete between-group separation. Odds ratios are presented with approximate 95% confidence intervals. Results are exploratory and require external validation. LA: left atrium; NT-proBNP: N-terminal pro-B-type natriuretic peptide; LAVI: left atrial volume index; MACE: major adverse cardiovascular events; CI: confidence interval; HF: heart failure

Variable	Odds ratio (OR)	95% CI	p-value
Baseline LA strain (%)	0.29	0.12-0.67	0.004
Atrial fibrillation	0.01	0.00-0.32	0.009
NT-proBNP (per 1000 pg/mL)	15.87	1.92-131.32	0.010
Baseline LAVI (per 5 mL/m²)	0.38	0.14-1.04	0.059
Age (per year)	0.97	0.82-1.14	0.676

**Figure 3:**
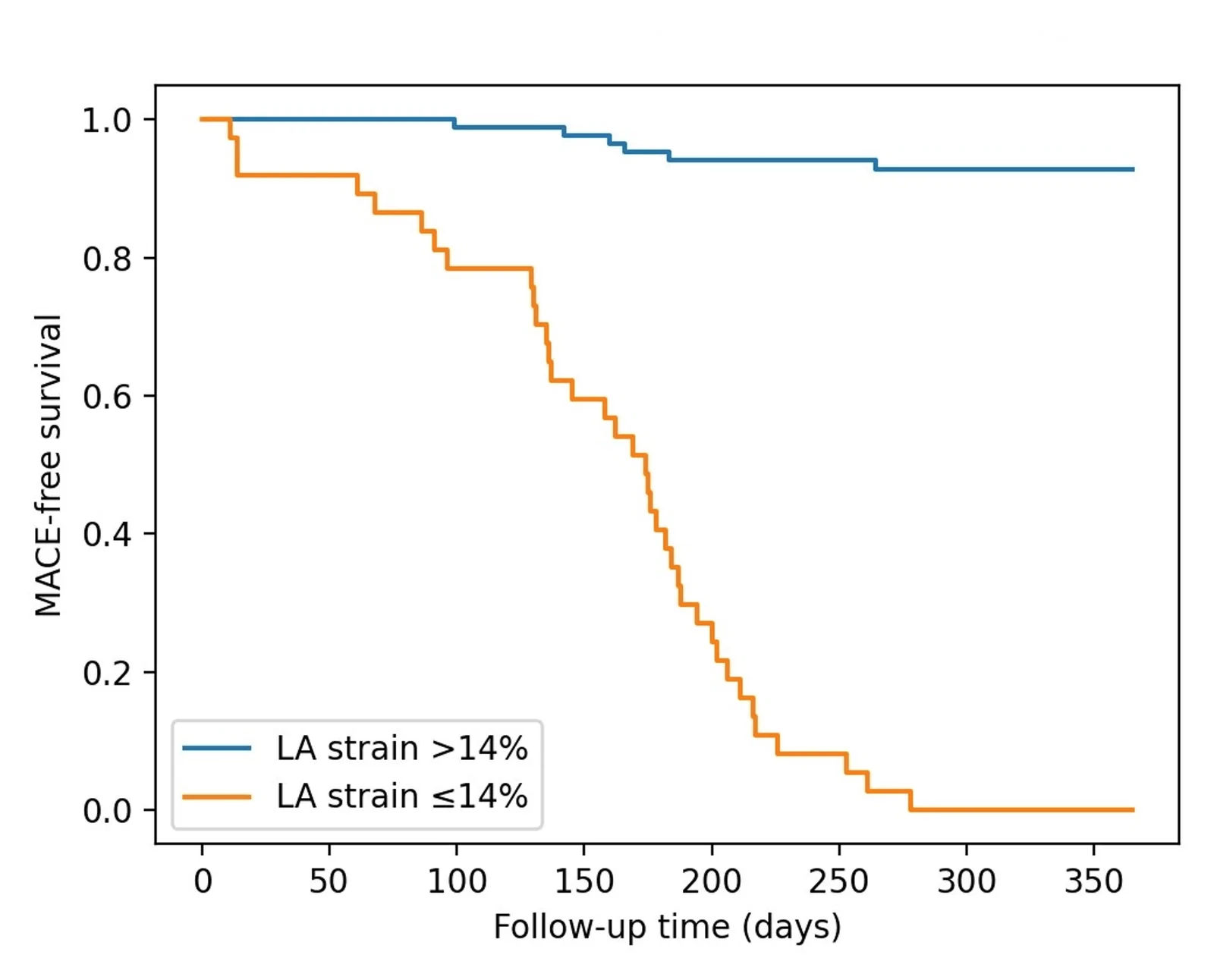
Kaplan-Meier curve by baseline LA strain Kaplan-Meier analysis of MACE-free survival according to baseline LA strain threshold ≤14%. Log-rank p < 0.001. LA: left atrium; MACE: major adverse cardiovascular events

Kaplan-Meier analysis demonstrated significantly lower MACE-free survival among patients with baseline LA strain ≤14.0% (log-rank p < 0.001; Figure 3).

## Discussion

The present study demonstrated a significant association between impaired left atrial (LA) strain and adverse clinical outcomes in patients hospitalized with acute decompensated HFpEF. Patients who experienced MACE had lower baseline and follow-up LA strain values together with less favorable echocardiographic and biomarker profiles. These findings suggest that LA strain reflects the severity of atrial remodeling and may complement conventional markers during risk assessment.

These findings are in agreement with the contemporary understanding of HFpEF as a multifactorial syndrome characterized by elevated filling pressures, myocardial remodeling, endothelial dysfunction, and progressive atrial dysfunction [5,6]. The LA appears to play an important role in HFpEF pathophysiology and may reflect the chronic burden of elevated filling pressures and diastolic dysfunction [7-10].

The LA contributes to left ventricular filling through reservoir, conduit, and booster pump functions. Chronic elevation of LV filling pressures leads to progressive LA remodeling, impaired compliance, and worsening reservoir function. LA strain directly evaluates this mechanical function and may detect atrial dysfunction earlier than LAVI, which primarily reflects structural remodeling [11-17].

Telles et al. demonstrated that patients with HFpEF had significantly impaired LA reservoir and pump strain, accompanied by increased LA stiffness, which correlated with elevated LV filling pressures and abnormal exercise hemodynamics. Furthermore, LA reservoir strain values below 33% showed good diagnostic performance for invasively confirmed HFpEF and improved noninvasive identification of elevated filling pressures compared with conventional echocardiographic parameters [25]. Although baseline E/e′ demonstrated slightly higher discriminative performance than baseline LA reservoir strain in our cohort, LA strain should be viewed as a complementary rather than competing parameter. It may provide additional information on atrial remodeling and dysfunction, particularly in patients with borderline or discordant conventional echocardiographic findings, thereby improving overall risk stratification.

In our cohort, LA strain was substantially impaired during index hospitalization. More importantly, patients with MACE failed to demonstrate meaningful recovery of LA strain during follow-up, while patients without MACE showed higher baseline and follow-up values. This observation suggests that the trajectory of atrial mechanical function may be clinically relevant and may reflect progression or stabilization of the HFpEF phenotype.

Our findings are consistent with previous studies demonstrating that impaired LA strain is associated with elevated filling pressures, abnormal exercise hemodynamics, reduced functional capacity, pulmonary hypertension, recurrent hospitalization, and mortality in HFpEF [23-27]. Contemporary imaging literature further emphasizes the role of LA strain in HFpEF phenotyping and atrial myopathy assessment [28-29].

Ye et al. showed that reduced LA reservoir strain was associated with elevated LV filling pressures during exercise, poorer functional capacity, and a higher likelihood of HFpEF in patients with dyspnea and preserved EF. They also reported that LA reservoir strain may provide diagnostic value when exercise testing is not available [26].

An important strength of the present analysis is the use of assessment of LA strain at baseline and one-year follow-up rather than a single baseline measurement. Most published studies have focused on baseline LA strain. By evaluating baseline and one-year follow-up values, the present study provides insight into dynamic atrial remodeling and its relationship with clinical outcomes.

Reduced LA strain was associated with several markers of advanced HFpEF, including elevated filling pressures, larger atrial volumes, pulmonary hypertension, and impaired ventricular mechanics. It reflects atrial myopathy, chronically elevated filling pressures, pulmonary vascular consequences, and ventricular-atrial coupling. A systematic review and meta-analysis further supported the prognostic value of LA strain in patients with heart failure [27].

From a clinical perspective, assessment of LA strain at baseline and one-year follow-up may help identify patients at increased risk of recurrent decompensation despite preserved LVEF. This could be particularly useful in patients with borderline or discordant conventional diastolic parameters, where LA strain may provide additional functional information. Reddy et al. reported that LA strain and compliance improve diagnostic assessment of HFpEF compared with conventional echocardiographic parameters alone [24].

Inclusion of patients with atrial fibrillation may have influenced LA strain measurements; interpretation of LA strain in atrial fibrillation remains methodologically challenging due to beat-to-beat variability and altered atrial mechanics, despite averaging measurements across multiple cardiac cycles. However, atrial fibrillation commonly coexists with HFpEF, and inclusion of these patients reflects routine clinical practice and improves real-world applicability of the study findings.

Reduced LA strain in the present cohort was accompanied by significantly elevated NT-proBNP levels, supporting the close pathophysiological relationship between atrial mechanical dysfunction and increased intracardiac filling pressures in HFpEF. NT-proBNP reflects myocardial wall stress and hemodynamic congestion, while impaired LA strain reflects chronic atrial remodeling, reduced compliance, and progressive atrial myopathy.

Combined assessment of LA strain and NT-proBNP may therefore improve risk stratification by integrating both acute hemodynamic stress and chronic atrial mechanical dysfunction. Assessment of LA strain at baseline and one-year follow-up could potentially identify patients with HFpEF at an increased risk of recurrent decompensation before overt deterioration of conventional echocardiographic parameters becomes evident.

Overall, the present findings support the potential clinical usefulness of assessment of LA strain at baseline and one-year follow-up as part of a comprehensive echocardiographic evaluation in HFpEF. However, larger prospective multicenter studies are required before routine implementation can be recommended.

Study limitations

This study has several limitations. First, it was conducted at a single tertiary center with a relatively modest sample size, which may limit the generalizability of the findings. Second, detailed information on baseline comorbidities and guideline-directed medical therapy was not systematically collected and therefore could not be included in the analysis. Third, although LA strain demonstrated good discriminatory performance for predicting adverse outcomes, the absence of external validation requires cautious interpretation of these findings. Therefore, larger prospective multicenter studies are needed to confirm the prognostic value of assessment of LA strain at baseline and one-year follow-up in patients with HFpEF.

## Conclusions

Reduced left atrial strain was associated with adverse one-year clinical outcomes in patients hospitalized with acute decompensated HFpEF. Assessment at two predefined time points of LA strain may provide complementary prognostic information when interpreted together with conventional echocardiographic parameters and NT-proBNP. These findings should be considered hypothesis-generating and require confirmation in larger prospective multicenter studies.

Introducing these parameters during regular echocardiographic assessment could contribute to a precise assessment of prognosis and the creation of an individual therapeutic protocol. However, due to the limitations of the study, these analyses should still be interpreted with caution and viewed as a basis for setting new research hypotheses that will be based on a larger, prospective multicenter study.
